# Tangled Troubles: A Rare Case of Rapunzel Syndrome in a Young Female Patient

**DOI:** 10.7759/cureus.90193

**Published:** 2025-08-15

**Authors:** Shivam Chauhan, Dheer S Kalwaniya, Madhur Gairola, B Harish Reddy

**Affiliations:** 1 General Surgery, Vardhman Mahavir Medical College and Safdarjung Hospital, New Delhi, IND

**Keywords:** ocd, rapunzel syndrome, trichobezoar, trichophagia, trichotillomania

## Abstract

Rapunzel syndrome is a rare and fascinating disease diagnosed when large trichobezoars are found in the patient's GI tract, and most of them suffer from obsessive-compulsive disorder (OCD) and trichotillomania. This report describes the case of a 27-year-old female patient who had abdominal pain, a progressively increasing abdominal mass, early satiety, and profound weight loss during the past six months. Physical examination, radiology, and endoscopy showed a large inhomogeneous mass filling the lumen from the stomach to the jejunum. Exploratory laparotomy, gastrotomy, and multiple enterotomies were done to remove a giant trichobezoar extending from the stomach to the transverse colon. The postoperative stay was uneventful, with the patient being allowed oral intake by day 3 and discharged on postoperative day (POD) 5 with proper psychiatric advice for continuation of antidepressants. Recognizing Rapunzel syndrome early can make the difference between a smooth recovery and life-threatening complications such as bowel obstruction or perforation. Timely surgery, followed by compassionate psychiatric care and ongoing support, is key not only to treating the immediate problem but also to helping patients avoid relapse and regain long-term well-being.

## Introduction

A trichobezoar is a hairball found in the proximal GI tract. It is an extremely rare entity, representing less than 1% of all bezoars, most commonly affecting adolescent females aged between 13 and 20 years, and is frequently associated with psychiatric conditions such as trichotillomania and trichophagia. In the early stage, most trichobezoars may go unnoticed due to vague symptoms or a complete lack of symptoms. It is important to consider trichobezoar in young females with psychiatric issues. This condition often arises from the desire to pull out one’s hair (trichotillomania) and swallow it (trichophagia). Other psychiatric disorders, such as pica, obsessive-compulsive disorder (OCD), depressive disorder, and anorexia nervosa, can also be linked with trichobezoar. In some instances, the trichobezoar can extend through the pylorus into the jejunum, ileum, or even colon. This situation, known as Rapunzel syndrome, was first described by Vaughan et al. in 1968 [1,2].

This syndrome typically involves large trichobezoars, or hair masses, forming in the GI tract due to hair consumption over months or years. According to Naik et al., to qualify all trichobezoar cases as Rapunzel syndrome, the following features are necessary:(1) a trichobezoar with a tail; (2) the tail extending at least to the jejunum; and (3) symptoms suggesting obstruction. Most reported cases involve females. This may be due to the traditional long hair of females, which increases the chances of entanglement and the formation of trichobezoar casts [3].

The clinical presentation can vary widely. Patients may experience abdominal pain, nausea, vomiting, abdominal distension, diarrhea or constipation, loss of appetite, weight loss, or an abdominal mass. A history of trichophagia is found in only 50% of patients. Complications from trichobezoars can involve GI obstruction, bleeding, perforation, malabsorption, and nutritional deficiencies. If left untreated, the mortality rate can reach 30% due to these complications [3,4].

Rapunzel syndrome is characterized by behavioral and mechanical changes. Chronic trichophagia leads to undigested hair building up in the stomach, where it mixes with mucus and food particles, forming a dense mass. This mass can grow into other parts of the gastrointestinal tract, causing the complications associated with Rapunzel syndrome [4].

This case report discusses a 27-year-old female patient with OCD and pica who developed a large abdominal mass and persistent abdominal pain over six months. Evaluation led to a diagnosis of Rapunzel syndrome and its subsequent management.

## Case presentation

A 27-year-old female patient presented with complaints of abdominal pain and vomiting in the emergency department with an enlarging abdominal mass over the last six months. She also had early satiety as well as significant weight loss during this period. Her symptoms had been exacerbated for the past two weeks. There was no history of non-passage of stool and flatus. She was recently diagnosed with OCD, for which she was taking fluoxetine. The patient denied any history of hair pulling and ingestion, but the parents claimed that the patient had a tendency to pull her hair and eat it, and also displayed pica, consuming non-nutritive substances such as dust and sand.

**Figure 1:**
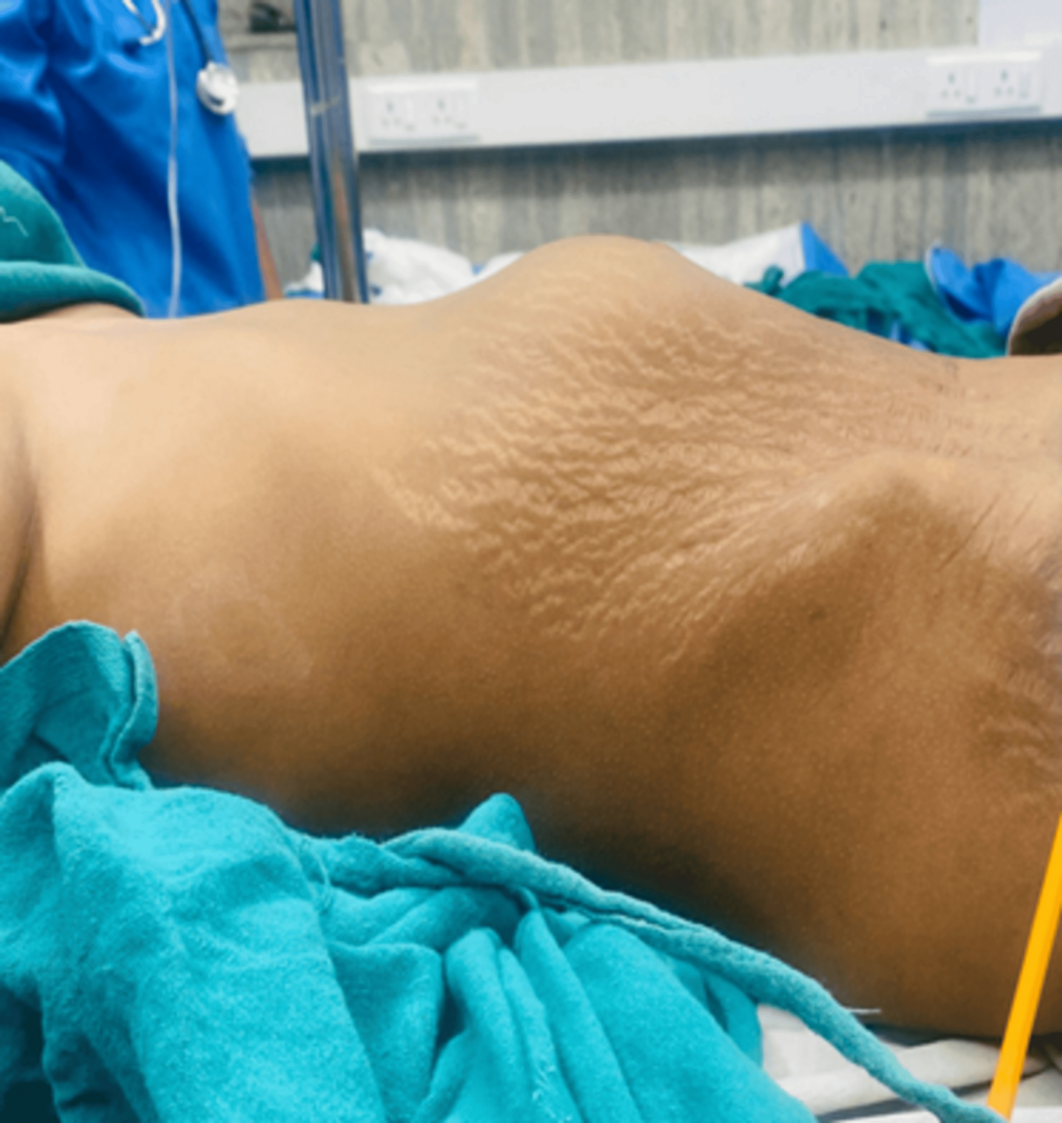
A globular-shaped mass from the lateral aspect was noted.

**Figure 2:**
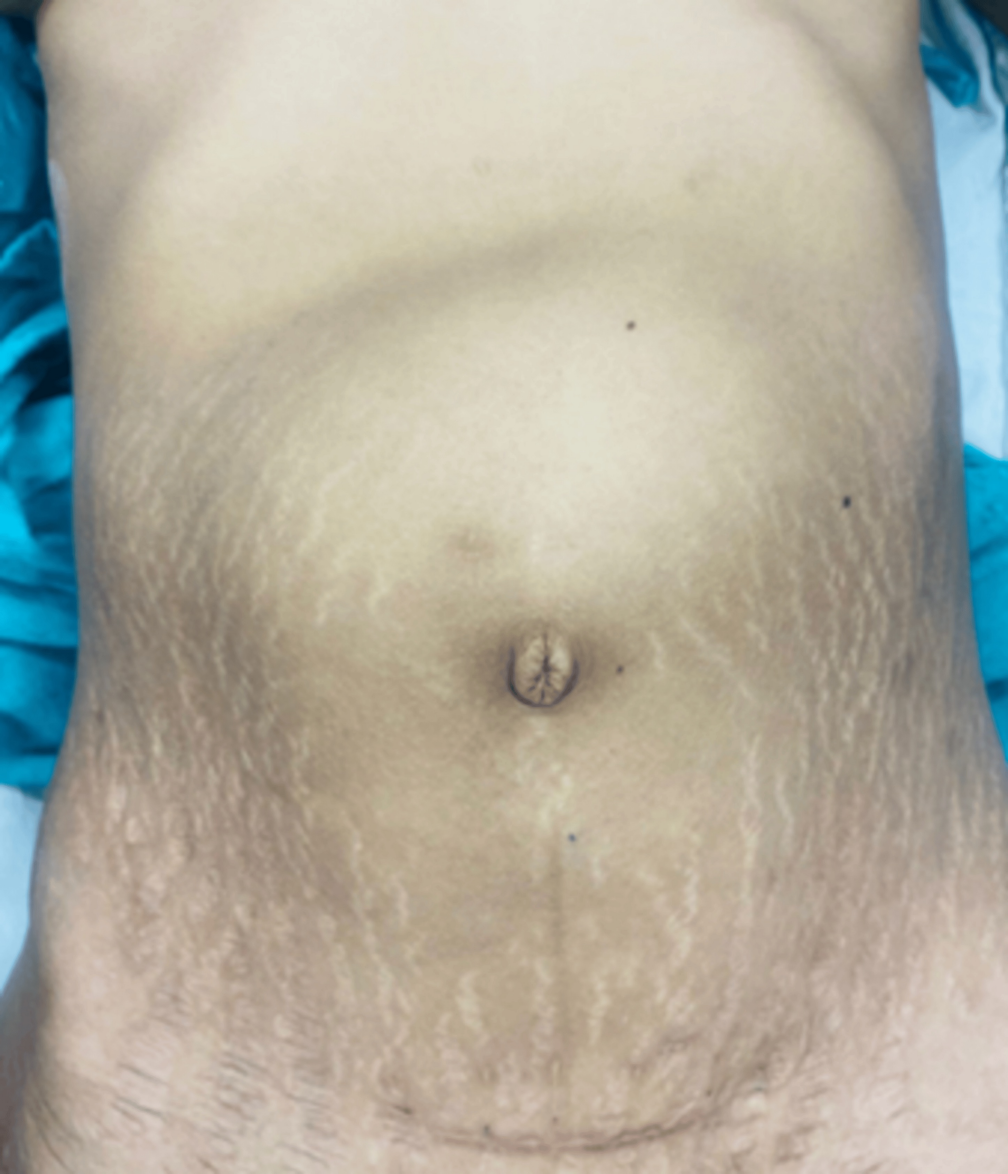
A globular-shaped mass was seen from the anterior view.

At presentation, the patient was cachectic with a BMI of 16 kg/m². Her vitals were normal at presentation. On physical examination, a round globular mass with a smooth surface was found in the upper abdomen (Figures 1, 2). On palpation, the mass was firm, immobile, and mildly tender. Bowel sounds were present, and the digital rectal examination was unremarkable. There were no signs of peritonitis.

**Figure 3:**
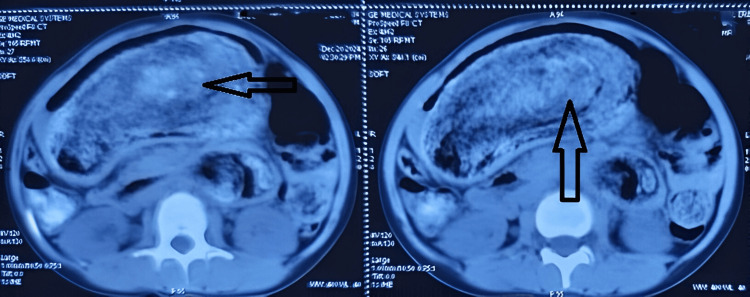
A contrast-enhanced CT (CECT) of the abdomen showed a trichobezoar in the stomach. The arrow shows a heterogeneous intraluminal mass with a mottled gas pattern in the lumen of the stomach.

A contrast-enhanced CT (CECT) of the abdomen was carried out, which showed the stomach was grossly distended, measuring up to the level of the umbilicus, and a heterogeneous intraluminal mass 16 x 15 x 10 cm in size with a mottled gas pattern in the lumen of the stomach reaching up to the pylorus, also extending into the duodenum and proximal jejunum at the left hypochondrium and left flank region (Figure 3). Mucosal thickening involving the stomach, duodenum, and proximal jejunum was noted. No obvious stricture or luminal narrowing was seen in small bowel loops.

**Figure 4:**
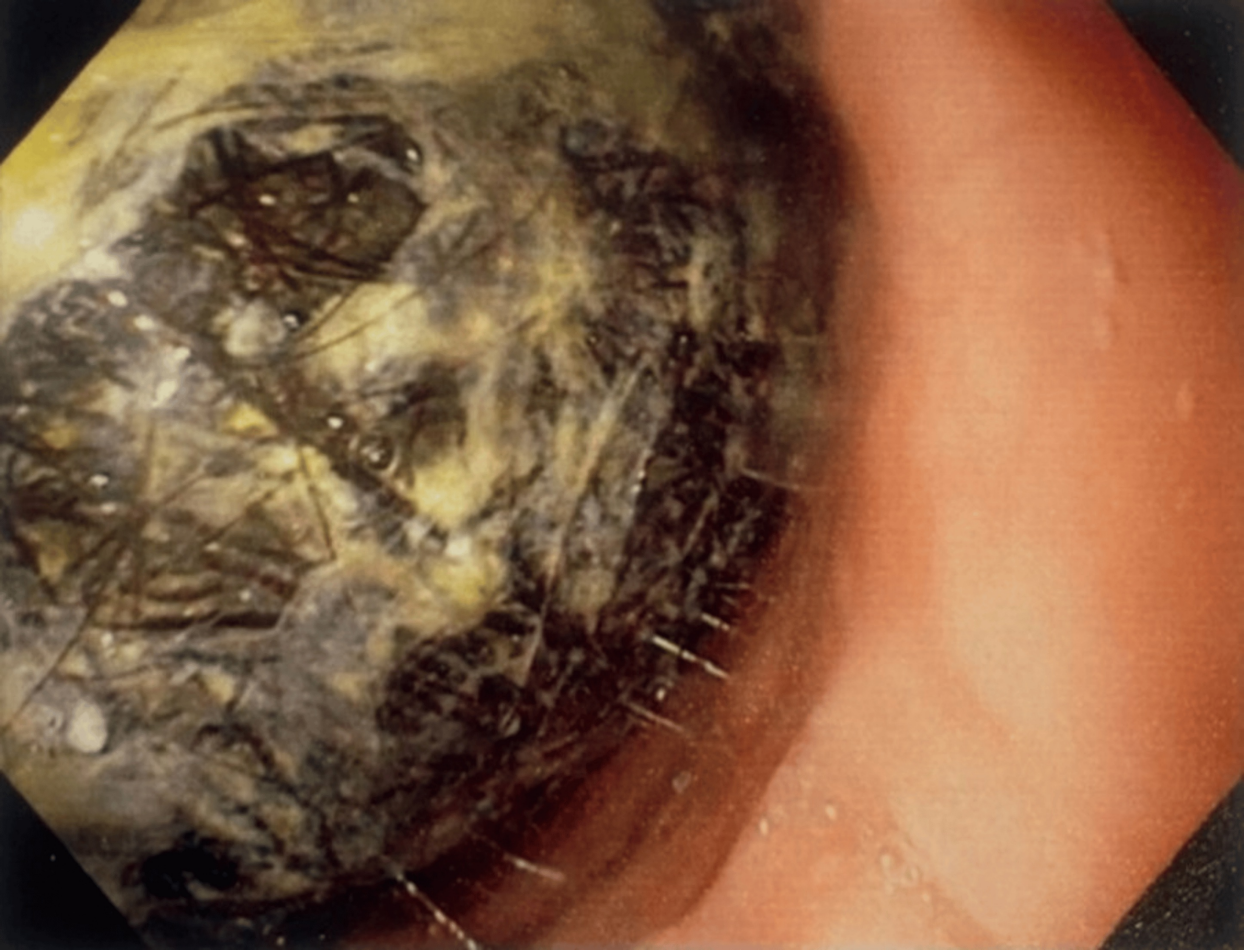
An endoscopic image showing a tuft of hair in the stomach.

Upper gastrointestinal endoscopy showed a large blackish mass of hair and fibrous material in the stomach occupying the fundus; the body and stomach scopes were not negotiable further. Gastric mucosa revealed a mild degree of erythema and edema of the mucosa (Figure 4).

The patient underwent an exploratory laparotomy. Intraoperative findings revealed a large trichobezoar extending from the stomach into the duodenum, jejunum, ileum, and even the transverse colon.

**Figure 5:**
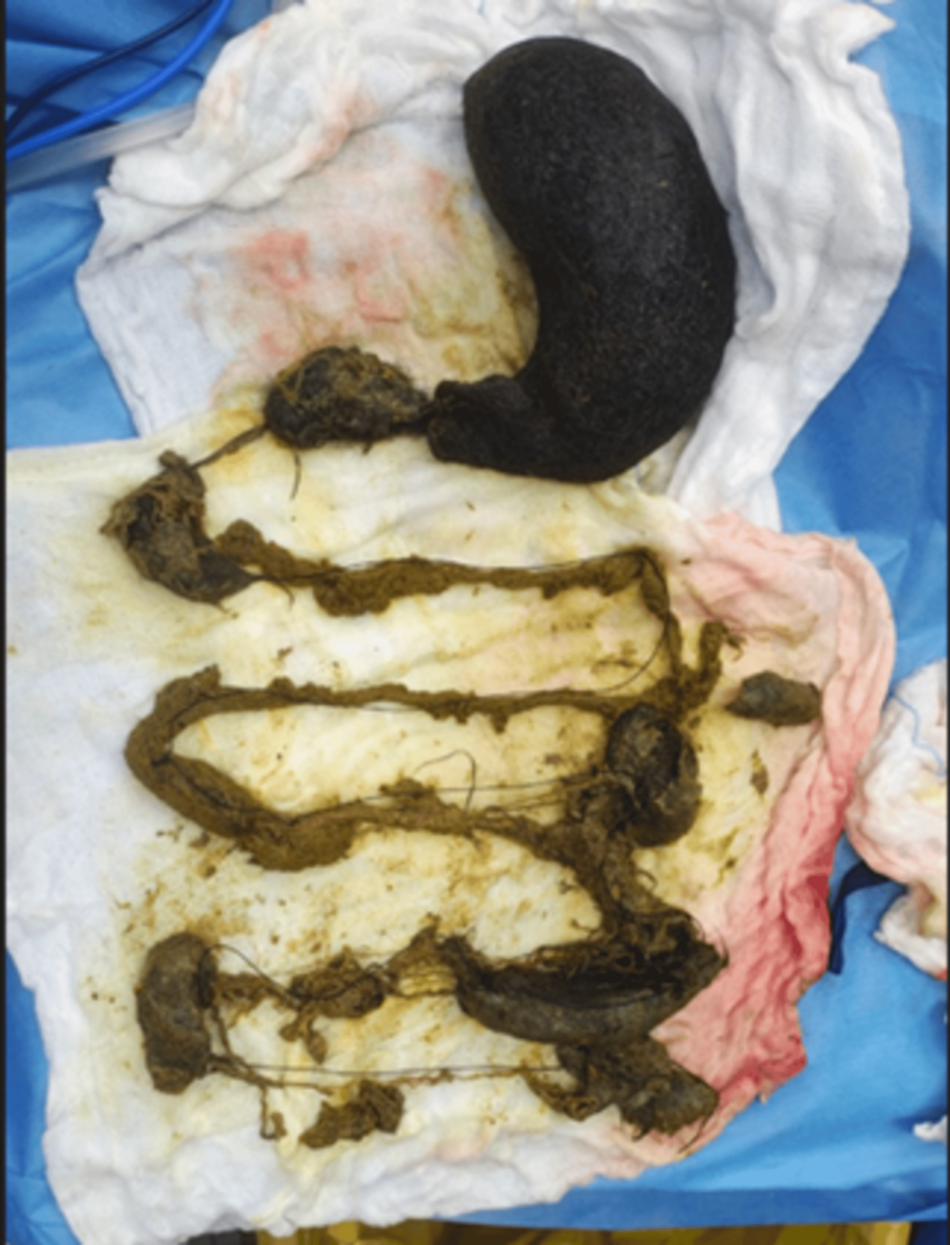
Postoperative trichobezoar specimen

A tuft of hair and threads was removed from the stomach and duodenum by gastrotomy. Jejunotomy was performed at the jejunum to remove the remaining trichobezoar, and then transverse colon enterotomy was done, which revealed hair and threads mixed with fecal matter, which were removed from the transverse colon. All gastrotomies and enterotomies were closed primarily (Figure 5).

In the postoperative period, oral sips were allowed, and the nasogastric tube was removed on postoperative day (POD) 3; the patient passed stool and flatus on POD 4, and she was discharged on POD 5. Overall, the postoperative period was uneventful. Psychiatric consultation was ordered, tab fluoxetine was restarted, and education for prevention of recurrence and nutritional support was planned. The patient is currently under regular follow-up and doing well.

## Discussion

Rapunzel syndrome is a rare and serious form of trichophagia, often leading to the formation of trichobezoars. This condition mainly affects young females with underlying mental health issues. In this case, the combination of OCD and pica likely led to a large bezoar. Bezoars are masses created by indigestible food or foreign materials in the gastrointestinal tract. Depending on their makeup, bezoars can be classified into several types: phytobezoars (made from vegetable or fruit fibers), trichobezoars (balls of hair or hair-like fibers), diospyrobezoars (from persimmon), pharmacobezoars (from pills), lactobezoars (from milk and curd), lithobezoars (fragments of stones), or plasticobezoars (plastic) [5]. Hair is slippery and can get stuck in the mucosal folds of the stomach lining, avoiding the normal movement that pushes food along. Over time, more hair collects and forms a mass shaped like a stomach. Mucus secreted in the stomach coats this trichobezoar, giving it a shiny surface. The stomach’s acid alters the hair protein, giving the bezoar a black color. The fermentation and breakdown of trapped food, especially fats, leads to a distinct rancid smell in the breath and the bezoar itself. Proline-rich proteins from the salivary glands help bind tannins and bezoar pieces together. Another factor in bezoar formation is reduced movement in the stomach.

Patients often remain without symptoms for many years. Symptoms typically begin to emerge as the trichobezoar grows large enough to cause blockage. The most common issues are related to upper GI obstruction, such as repeated vomiting, lack of appetite, weight loss, and an abdominal lump upon examination. Indirect signs from malabsorption can include iron deficiency leading to microcytic and hypochromic anemia, vitamin B12 deficiency causing megaloblastic anemia, fatigue, loss of protein through the gut, and weight loss. A large, obstructing, or eroding bezoar may lead to complications like gastric ulcers, obstructive jaundice, and acute pancreatitis [6].

The diagnostic process, which includes imaging and endoscopy, is very important for evaluating the extent of gastrointestinal involvement. Upper GI endoscopy is considered the gold standard for diagnosing trichobezoars. This procedure shows a mass made of hair, which appears black due to the gastric acid’s effect on the hair protein, mixed with mucus and food. However, the most frequently used imaging tool reported in the literature is CT, which reveals a well-defined intraluminal, oval-shaped mass with gas pockets. Surgery is the main treatment for large bezoars. Other methods, such as extracorporeal shock wave lithotripsy, enzyme treatments (like pancreatic lipase and cellulose), and medications (like metoclopramide and acetylcysteine), show varied success rates.

The endoscopic method may work well for phytobezoars or lactobezoars since they are usually smaller. Specialized devices, like bezotomes and bezotriptors, are used to break up solid trichobezoars [7]. Our patient underwent a laparotomy, and the large trichobezoar and its tail were successfully removed with the help of a gastrotomy and multiple enterotomies. The long-term outlook for these patients fully depends on preventing recurrences. Therefore, parental counseling, behavioral therapy to control trichotillomania and trichophagia, psychiatric support, psychological help, and ongoing follow-up are crucial for improving the outcomes for patients diagnosed with trichobezoars. Additionally, we started treatment with fluoxetine in our case after surgery. However, it’s essential to address the underlying mental health condition to prevent recurrences.

The prevention of recurrence in trichobezoar cases hinges on effectively treating the underlying trichotillomania and trichophagia. Traditionally, behavioral interventions such as habit reversal training (HRT), metacognitive therapy combined with HRT, and dialectical behavior therapy (DBT)-enhanced HRT have shown the most consistent evidence for reducing hair-pulling behaviors, often outperforming pharmacological approaches such as SSRIs or clomipramine in effect size [8-10].

Recently, tele-based HRT has emerged as a promising and accessible modality, particularly valuable in pediatric populations and in regions where specialized therapy is not widely available. This approach enables remote delivery of structured behavioral interventions, improving adherence and continuity of care while maintaining comparable efficacy to in-person therapy [11].

Incorporating such evidence-based psychiatric and behavioral management into the post-surgical care of trichobezoar patients, along with parental counseling, psychological support, and regular follow-up, offers the best chance of preventing recurrence and improving long-term outcomes.

## Conclusions

This case underscores the importance of a multidisciplinary approach in diagnosing and managing Rapunzel syndrome. Early recognition of behavioral symptoms and timely surgical intervention can improve outcomes in patients with this rare condition. Further research is needed to better understand the pathophysiology and optimal management strategies for Rapunzel syndrome.
